# Transmurality mapping of left ventricular scar: impact of spatial resoluton

**DOI:** 10.1186/1532-429X-13-S1-P163

**Published:** 2011-02-02

**Authors:** Dana C Peters, Felix Liu, Alex Tan, Benjamin R Knowles, Heather S Duffy, Andrew L Wit, Mark E Josephson, Warren J Manning

**Affiliations:** 1 Beth Israel Deaconess Medical Center, Boston, MA, USA; 2 Columbia University, New York, NY, USA

## Purpose

To generate scar transmurality maps, to study the impact of spatial resolution on these maps.

## Introduction

Left ventricular (LV) scar is associated with ventricular tachycardia (VT), related to regions of slow conduction. Endocardial LV voltage maps reveal regions of low voltage near the site of VT origin, corresponding to myocardial fiber bundles trapped in areas of extensive fibrosis. Scar transmurality (i.e. the percent of myocardial wall thickness which is scarred, measured in 6-12 sectors around the LV myocardium) is an indicator of myocardial viability after reperfusion (1). Intermediate infarct transmurality has been correlated with non-ischemic VT (2). Furthermore, lower voltages are found in regions of more transmural scarring (3). We have expanded the transmurality concept to generate LGE transmurality maps of the LV.

## Methods

**Figure 1:**
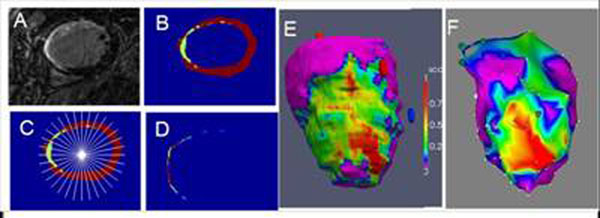
A) Starting with a short axis LGE image (A), scar and myocardium are segmented (B). Scar transmurality is calculated in 100 sectors in each slice (C), which provides an endocardial representation of LV scar (D). E) Scar transmurality map (1= 100%) transmural) is displayed in 3D. F) The corresponding bipolar voltage maps shows strong similarity (scale=0.5/0.05mV).

3D LGE studies (4) from 8 patients with prior myocardial infarction were obtained on a 1.5T Achieva CMR system (Philips Healthcare, Best, NL). Imaging parameters were: 0.2mmol/kg Gd-DPTA, inversion recovery gradient echo sequence with fat-saturation, with ECG and NAV-gating: TR/TE/θ=5.6ms/2.6ms/25°, 4-6 minutes/scan. Figure 1 illustrates the processing to generate 3D endocardial transmurality maps, using original high (1.3 x 1.3 x 4mm^3^) and processed lower (2 x 2 x 8mm^3^) resolution images. All processing was performed in Matlab (v7.1, Mathworks, Natick, MA), with visualization using Paraview (v3.8 Kitware, Clifton Park, NY). Bipolar voltage maps, obtained by electrophysiological (EP) catheter mapping, were compared with transmurality maps visually. High and low resolution transmurality maps were compared visually. Average transmurality of scarred sectors was compared, using a paired t-test.

## Results

**Figure 2:**
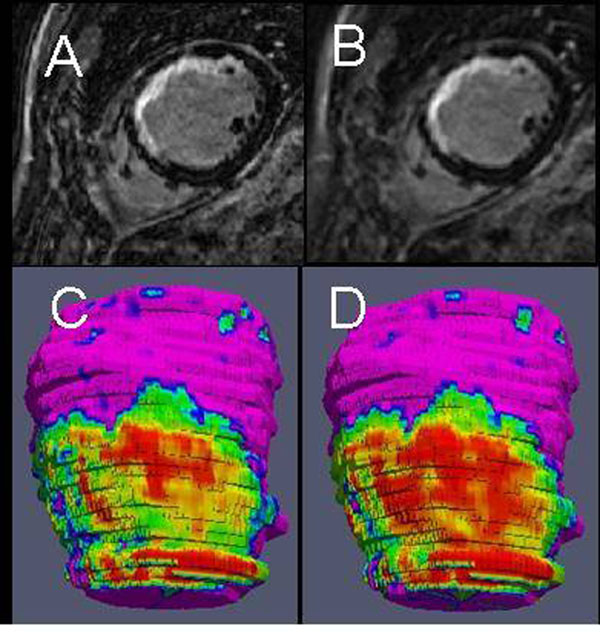
Example higher (A) and lower (B) resolution LGE images, and respective transmurality maps from higher (C) and lower (D) resolution images.

In 4 subjects with voltage maps, regions with greater transmurality had lower voltage electrograms (Fig 1E,F). Low resolution data produced greater transmurality by visual assessment in 88% of subjects (Figure 2). Quantitatively, the average scar transmurality in scarred sectors for high and low resolution transmurality maps were 30 ±4% and 37±5%, p=0.001, respectively.

## Conclusions

We present a novel method for displaying transmurality of enhancement from 3D LGE images. The relationship between transmurality maps and voltage must be further in explored in a larger cohort, using quantitative means. Maps derived from higher resolution images demonstrated less transmural scar. Since scar that encompasses surviving myocardium (i.e. almost transmural scar) may be related to VT genesis, high resolution transmurality mapping might be useful in detecting the true arrhythmogenic substrate.
